# Trends and Disparities in Glycemic Control and Severe Hyperglycemia Among US Adults With Diabetes Using Insulin, 1988-2020

**DOI:** 10.1001/jamanetworkopen.2022.47656

**Published:** 2022-12-20

**Authors:** Siddharth Venkatraman, Justin B. Echouffo-Tcheugui, Elizabeth Selvin, Michael Fang

**Affiliations:** 1 Johns Hopkins University School of Medicine, Baltimore, Maryland; 2 Welch Center for Prevention, Epidemiology, and Clinical Research, Department of Epidemiology, Johns Hopkins Bloomberg School of Public Health, Baltimore, Maryland

## Abstract

**Importance:**

There have been major advances in insulin delivery and formulations over the past several decades. It is unclear whether these changes have resulted in improved glycemic control for patients with diabetes.

**Objective:**

To characterize trends and disparities in glycemic control and severe hyperglycemia in US adults with diabetes using insulin.

**Design, Setting, and Participants:**

This serial population-based cross-sectional study used data from the National Health and Nutrition Examination Survey (NHANES) between 1988-1994 and 1999-2020. Participants were nonpregnant US adults aged 20 years or older who had a diagnosis of diabetes and were currently using insulin.

**Exposures:**

Diabetes diagnosis and use of insulin.

**Main Outcomes and Measures:**

Trends in glycemic control (glycated hemoglobin [HbA_1c_] level <7%) and severe hyperglycemia (HbA_1c_ level >10%; to convert percentage of total hemoglobin to proportion of total hemoglobin, multiply by 0.01; to convert to millimoles per mole, multiply by 10.93 and subtract by 23.50) overall and by age, race and ethnicity, and indicators of socioeconomic status were evaluated using logistic regression. Analyses incorporated sample weights to account for oversampling of certain populations and survey nonresponse.

**Results:**

There were 2482 participants with diabetes using insulin included in the analyses (mean [SD] age, 59.8 [0.4] years); 51.3% were men, 7.0% were Mexican American individuals, 17.9% were non-Hispanic Black individuals, and 65.2% were non-Hispanic White individuals. From 1988-1994 to 2013-2020, the proportion of patients with diabetes who received insulin and achieved glycemic control did not significantly change, from 29.2% (95% CI, 22.6%-36.8%) to 27.5% (95% CI, 21.7%-34.2%). Mexican American adults who received insulin were less likely than non-Hispanic White adults to achieve glycemic control, and disparities increased during the study period. The proportion of adults with severe hyperglycemia did not significantly change and was 14.6% (95% CI, 12.0-17.5) in 2013-2020. Adults who were Mexican American or non-Hispanic Black, were uninsured, or had low family income had the highest prevalence of severe hyperglycemia.

**Conclusions and Relevance:**

In this population-based cross-sectional study of NHANES data over the past 3 decades, glycemic control stagnated and racial and ethnic disparities increased among US adults with diabetes who received insulin. Efforts to improve access to insulin may optimize glycemic control and reduce disparities in this population.

## Key Points

**Question:**

Has glycemic control improved among US adults with diabetes using insulin over the past 30 years?

**Findings:**

In this cross-sectional study of 2482 US adults with diabetes using insulin, the prevalence of glycemic control (glycated hemoglobin level <7%) remained unchanged (29.2% in 1988-1994 to 27.5% in 2017-2020). Mexican American adults using insulin were less likely than non-Hispanic White adults to achieve glycemic control, and disparities increased during the study period.

**Meaning:**

This study found that over the past 3 decades, glycemic control stagnated and racial and ethnic disparities increased among US adults with diabetes using insulin.

## Teaser

This cross-sectional study uses data from the National Health and Nutrition Examination Survey to evaluate trends in glycemic control and severe hyperglycemia among US patients diagnosed with diabetes using insulin from 1988 to 2020.

## Introduction

Insulin is typically a last-line therapy for patients with type 2 diabetes. Over the past several decades, there have been major advances in diabetes technology and management strategies as well as insulin delivery and formulations.^1,2,3^ However, little is known regarding recent patterns of glycemic control (glycated hemoglobin [HbA_1c_] level <7%; to convert percentage of total hemoglobin to proportion of total hemoglobin, multiply by 0.01; to convert to millimoles per mole, multiply by 10.93 and subtract by 23.50) among patients using insulin. Additionally, few studies have examined severe hyperglycemia (defined as HbA_1c_ level >10%), which insulin therapy specifically aims to address.^4^ Understanding these trends can inform health policy and public health initiatives to improve glycemic control in patients receiving insulin.

Racial and ethnic minority patients, persons from low–socioeconomic status backgrounds, and those without insurance experience slower intensification of their treatment regimen and have less access to technologies that improve the safety of insulin therapy (eg, continuous glucose monitoring systems), potentially contributing to worse glycemic control.^5^ In addition, insulin prices have tripled in the US, while out-of-pocket costs per prescription doubled over the past decade.^6^ However, few studies have examined how these disparities affect glycemic control over time, specifically among patients with diabetes using insulin. Characterizing population-level disparities is important for designing policies and targeted interventions to address inequities among patients receiving insulin.

The objective of our study was to characterize national trends in glycemic control and severe hyperglycemia among patients with diagnosed diabetes using insulin. We considered these outcomes overall and by race and ethnicity, educational level, income, and health insurance status. To accomplish these objectives, we conducted an analysis of over 3 decades of data (1988-2020) from the National Health and Nutrition Examination Survey (NHANES).

## Methods

### Study Design

In this cross-sectional study, we analyzed data from the NHANES III, which was conducted from 1988 to 1994, and from the continuous NHANES, with data available from 1999 to 2020. The NHANES uses a stratified, multistage, probability-cluster design to ensure that sample populations are representative of the nation’s noninstitutionalized civilians. Data are collected from household interviews and from standardized medical examinations including blood sample collections performed in mobile examination centers. Our analysis included nonpregnant adults aged 20 years or older who reported a diagnosis of diabetes by a doctor or health professional other than during pregnancy and who were currently being treated with insulin. The National Center for Health Statistics institutional review board approved the study protocols, and all the participants provided written informed consent. This study followed the Strengthening the Reporting of Observational Studies in Epidemiology (STROBE) reporting guideline.^7^

### Glycemic Control and Severe Hyperglycemia

Level of HbA_1c_ was measured with the use of high-performance liquid chromatography. To account for changing laboratory methods over time, we calibrated HbA_1c_ levels using a previously validated equipercentile equating approach to correct for shifts in distribution due to laboratory drift.^8^ Glycemic control was defined as an HbA_1c_ level less than 7% (53 mmol/mol).^4^ Severe hyperglycemia was defined as an HbA_1c_ level greater than 10% (86 mmol/mol), an HbA_1c_ threshold that is a typical indication for insulin initiation to prevent diabetes complications.^4^ In sensitivity analyses, we also considered alternative definitions of glycemic control (HbA_1c_ level <8% [64 mmol/mol]) and severe hyperglycemia (HbA_1c_ level >9% [75 mmol/mol]).^9^ Sensitivity analyses were also conducted using age-adjusted estimates and 2 age-specific cutoff models: (1) HbA_1c_ level less than 7% for nonpregnant adults and HbA_1c_ level less than 8% for patients older than 75 years and (2) HbA_1c_ level less than 7% for nonpregnant adults younger than 65 years, HbA_1c_ level less than 7.5% (58 mmol/mol) for adults aged 65 to 74 years, and HbA_1c_ level less than 8% for adults aged 75 years or older.^10,11^

### Sociodemographic and Other Measures

Participants self-reported their age, gender (man or woman), race and ethnicity (Mexican American, non-Hispanic Black, non-Hispanic White, or other race and ethnicity), educational level (high school or less, some college, or college graduate or above), health insurance status (uninsured, private insurance, or public insurance), health care utilization (number of visits to a health care professional annually), age at diabetes diagnosis, and family income. Based on family income, the income-poverty ratio was categorized as less than 130% of the federal poverty level (FPL), 130% to 349% of the FPL, or 350% or more of the FPL. Body mass index (calculated as weight in kilograms divided by height in meters squared) was calculated from measured height and weight and categorized as less than 25, 25 to less than 30, or 30 or greater.^12^

### Statistical Analyses

We used χ^2^ and *t* tests to assess differences in sociodemographic and clinical characteristics among adults using insulin. We estimated trends in insulin use, glycemic control, and severe hyperglycemia overall and by age, race and ethnicity, and indicators of socioeconomic status. To increase the precision of our point estimates, we pooled NHANES survey cycles into 5- to 7-year intervals (1988-1994, 1999-2004, 2005-2012, and 2013-2020). We used logistic regression to evaluate trends over time with the midpoint of each survey cycle modeled as a continuous independent variable.^13^ We also assessed likelihood of achieving glycemic control or having severe hyperglycemia after adjustment for age, gender, race and ethnicity, educational level, and income-poverty ratio using logistic regression models. We conducted sensitivity analyses (1) using different uniform HbA_1c_ cutoff values of glycemic control and severe hyperglycemia, (2) using age-adjusted and age-specific HbA_1c_ cutoff values, and (3) excluding persons with possible type 1 diabetes, defined as those who started using insulin within 1 year of diabetes diagnosis, were currently using insulin, and were diagnosed with diabetes when younger than 30 years.^14^

All analyses were conducted using Stata, version 17.0 (StataCorp LLC) and incorporated the recommended sample weights to account for oversampling of certain populations and survey nonresponse.^15^ We calculated the variance of estimates using recommended Taylor Series linearization procedures on masked variance units provided on the demographic data files. The calculated estimates are designed to be representative of the US civilian noninstitutionalized population with diagnosed diabetes. We used 2-sided *P* < .05 as an indicator of statistical significance.

## Results

### Characteristics of US Adults With Diabetes Using Insulin

The demographic profile for the 2482 participants is summarized in Table 1. These participants had a mean (SD) age of 59.8 (0.4) years; 51.3% were men, 48.7% were women, 7.0% were Mexican American individuals, 17.9% were non-Hispanic Black individuals, and 65.2% were non-Hispanic White individuals. The overall percentage of adults with diabetes who used insulin did not change significantly, from 30.5% in 1988-1994 to 28.2% in 2013-2020 (*P* = .81 for trend) (eTable 1 in Supplement 1).

**Table 1.** Characteristics of US Adults Diagnosed With Diabetes Using Insulin in NHANES From 1988 to 2020

Characteristic	Participants (N = 2482)^a^			
	1988-1994 (n = 475)	1999-2004 (n = 388)	2005-2012 (n = 755)	2013-2020 (n = 864)
Age, mean, y	60.1 (56.7-63.4)	59.2 (57.0-61.4)	58.9 (57.6-60.3)	60.6 (59.2-61.9)
Age category, y				
20-49	24.8 (15.7-36.9)	23.8 (18.2-30.5)	23.8 (19.9-28.1)	21.5 (18.2-25.3)
50-64	29.0 (23.0-35.9)	33.5 (27.4-40.2)	38.6 (34.3-43.0)	35.6 (31.2-40.3)
≥65	46.2 (38.7-53.8)	42.7 (35.9-49.6)	37.6 (33.5-42.0)	42.8 (37.9-47.9)
Gender				
Men	41.7 (33.4-50.6)	45.4 (38.9-52.1)	53.4 (49.1-57.6)	54.9 (49.7-60.0)
Women	58.3 (49.4-66.6)	54.6 (47.9-61.1)	46.6 (42.4-50.9)	45.1 (40.0-50.3)
Race and ethnicity^b^				
Mexican American	4.2 (3.1-5.7)	5.0 (3.2-7.9)	6.5 (4.2-10.1)	9.0 (6.5-12.3)
Non-Hispanic Black	21.5 (16.3-27.8)	19.6 (13.4-27.7)	19.4 (15.7-23.9)	14.9 (11.9-18.5)
Non-Hispanic White	70.8 (63.4-77.2)	65.8 (57.8-73.1)	65.6 (59.3-71.3)	63.2 (57.9-68.2)
BMI				
Mean	30.1 (29.1-31.1)	32.2 (30.9-33.5)	33.6 (32.8-34.4)	33.4 (32.6-34.1)
<25	24.4 (18.5-31.5)	16.6 (11.3-23.8)	13.0 (10.1-16.5)	9.0 (6.8-11.9)
25 to <30	37.3 (28.7-46.9)	22.9 (18.3-28.1)	20.5 (16.7-25.0)	24.4 (19.7-29.7)
≥30	38.3 (31.3-45.8)	60.5 (53.1-67.5)	66.5 (61.9-70.8)	66.6 (61.1-71.7)
Educational level				
College graduate or above	8.3 (5.4-12.5)	13.3 (8.9-19.4)	17.3 (13.2-22.2)	16.7 (13.4-20.5)
Some college	15.3 (7.8-27.9)^c^	23.6 (17.6-30.7)	31.6 (27.4-36.2)	33.0 (28.8-37.4)
High school or less	76.4 (63.4-85.9)	63.1 (56.4-69.4)	51.1 (45.7-56.5)	50.3 (45.5-55.2)
Insurance status				
Insured	93.6 (87.7-96.7)	91.9 (86.4-95.3)	90.3 (86.6-93.0)	95.6 (93.9-96.9)
Private insurance	69.7 (59.5-78.3)	57.3 (50.2-64.1)	56.3 (50.9-61.4)	53.6 (48.4-58.6)
Public or other insurance	23.8 (17.3-31.9)	34.6 (29.5-40.0)	34.0 (29.6-38.7)	42.0 (37.2-47.1)
Uninsured	6.4 (3.3-12.3)^c^	8.1 (4.7-13.6)	9.7 (7.0-13.4)	4.4 (3.1-6.1)
Visits to a doctor or clinic in the past year, No.				
0	2.3 (1.1-5.1)^c^	0.8 (0.1-4.5)^c^	2.4 (0.9-6.6)^c^	1.2 (0.6-2.2)^c^
1	6.6 (3.6-12.0)^c^	3.4 (1.6-7.1)^c^	3.1 (1.7-5.6)	3.0 (1.7-5.1)
2-3	20.6 (13.3-30.6)	15.9 (11.2-22.2)	16.0 (12.8-19.8)	19.4 (15.5-24.1)
≥4	70.4 (61.9-77.7)	79.9 (73.5-85.0)	78.5 (74.3-82.2)	76.4 (71.6-80.6)
Immigration status				
US-born	94.8 (91.2-96.9)	90.1 (86.0-93.0)	91.2 (88.8-93.1)	85.2 (81.8-88.0)
Not US-born	5.2 (3.1-8.8)	9.9 (7.0-14.0)	8.8 (6.9-11.2)	14.8 (12.0-18.2)
Income-poverty ratio				
Mean	2.5 (2.1-3.0)	2.5 (2.2-2.7)	2.6 (2.5-2.8)	2.7 (2.4-2.9)
<130% of FPL	26.0 (19.7-33.4)	27.3 (21.7-33.7)	23.9 (20.2-28.0)	27.5 (23.0-32.5)
130%-349% of FPL	42.8 (35.1-50.9)	36.5 (30.6-42.8)	37.9 (32.9-43.1)	32.6 (27.1-38.6)
≥350% of FPL	31.2 (23.5-40.3)	36.2 (29.5-43.5)	38.3 (33.8-43.0)	39.9 (32.8-47.3)
Calibrated HbA_1c_ level, %	8.2 (7.9-8.5)	8.2 (7.9-8.5)	8.0 (7.8-8.2)	8.1 (7.9-8.3)
Duration of diabetes, mean, y	12.9 (11.5-14.3)	17.7 (15.7-19.7)	17.1 (15.9-18.2)	17.8 (16.8-18.8)

Abbreviations: BMI, body mass index (calculated as weight in kilograms divided by height in meters squared); FPL, federal poverty level; HbA_1c_, glycated hemoglobin; NHANES, National Health and Nutrition Examination Survey.

SI conversion factor: To convert percentage of total hemoglobin to proportion of total hemoglobin, multiply by 0.01; to convert to millimoles per mole, multiply by 10.93 and subtract by 23.50.

^a^ Sample sizes are unweighted. Data are presented as weighted percentage (95% CI) unless otherwise indicated.

^b^ Adults who responded “other” for race and ethnicity were excluded in the race and ethnicity analysis but were included for all other subgroup analyses.

^c^ Estimate has a relative SE of 30% or greater and may be unreliable.

Among adults using insulin for their diabetes treatment, the current mean age was 60.6 years (95% CI, 59.2-61.9 years) and did not change significantly over time (*P* = .39 for trend) (Table 1). From 1988-1994 to 2013-2020, there was a significant increase in mean diabetes duration (12.9 years [95% CI, 11.5-14.3 years] to 17.8 years [95% CI, 16.8-18.8 years]; *P* < .001 for trend) as well as the proportion of individuals who were Mexican American (4.2% [95% CI, 3.1%-5.7%] to 9.0% [95% CI, 6.5%-12.3%]; *P* = .003 for trend), covered by public or other insurance (23.8% [95% CI, 17.3%-31.9%] to 42.0% [95% CI, 37.2%-47.1%]; *P* = .001 for trend), and had a body mass index greater than 30 (38.3% [95% CI, 31.3%-45.8%] to 66.6% [95% CI, 61.1%-71.7%]; *P* = .02 for trend).

### Trends in Glycemic Control and Severe Hyperglycemia

**Figure 1.**
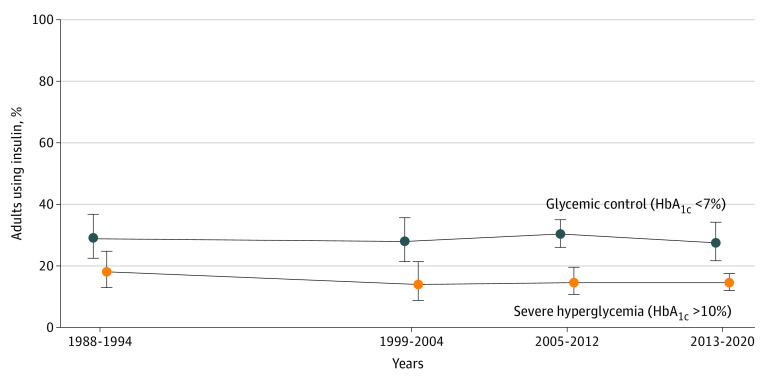
Trends in Glycemic Control and Severe Hyperglycemia Among US Adults With Diabetes Using Insulin From 1988 to 2020 Data are from the 1988 to 2020 National Health and Nutrition Examination Survey (N = 2482) and were weighted to be nationally representative. Glycemic control was defined as a glycated hemoglobin (HbA_1c_) level less than 7%, and severe hyperglycemia as an HbA_1c_ level greater than 10% (to convert percentage of total hemoglobin to proportion of total hemoglobin, multiply by 0.01; to convert to millimoles per mole, multiply by 10.93 and subtract by 23.50). Error bars indicate 95% CIs.

From 1988-1994 to 2013-2020, there was no significant change in the proportion of adults using insulin who achieved glycemic control (HbA_1c_ level <7%) (29.2% [95% CI, 22.6%-36.8%] to 27.5% [95% CI, 21.7%-34.2%]; *P* = .87 for trend) or had severe hyperglycemia (HbA_1c_ level >10%) (18.2% [95% CI, 13.0%–24.8%] to 14.6% [95% CI, 12.0%-17.5%]; *P* = .28 for trend) (Figure 1). The mean HbA_1c_ level from 1988-2020 was 8.1% (95% CI, 8.0%-8.2%; 65 mmol/mol [95% CI, X.X-X.X mmol/mol) and did not change significantly over time (*P* = .57 for trend) (Table 1).

**Figure 2.**
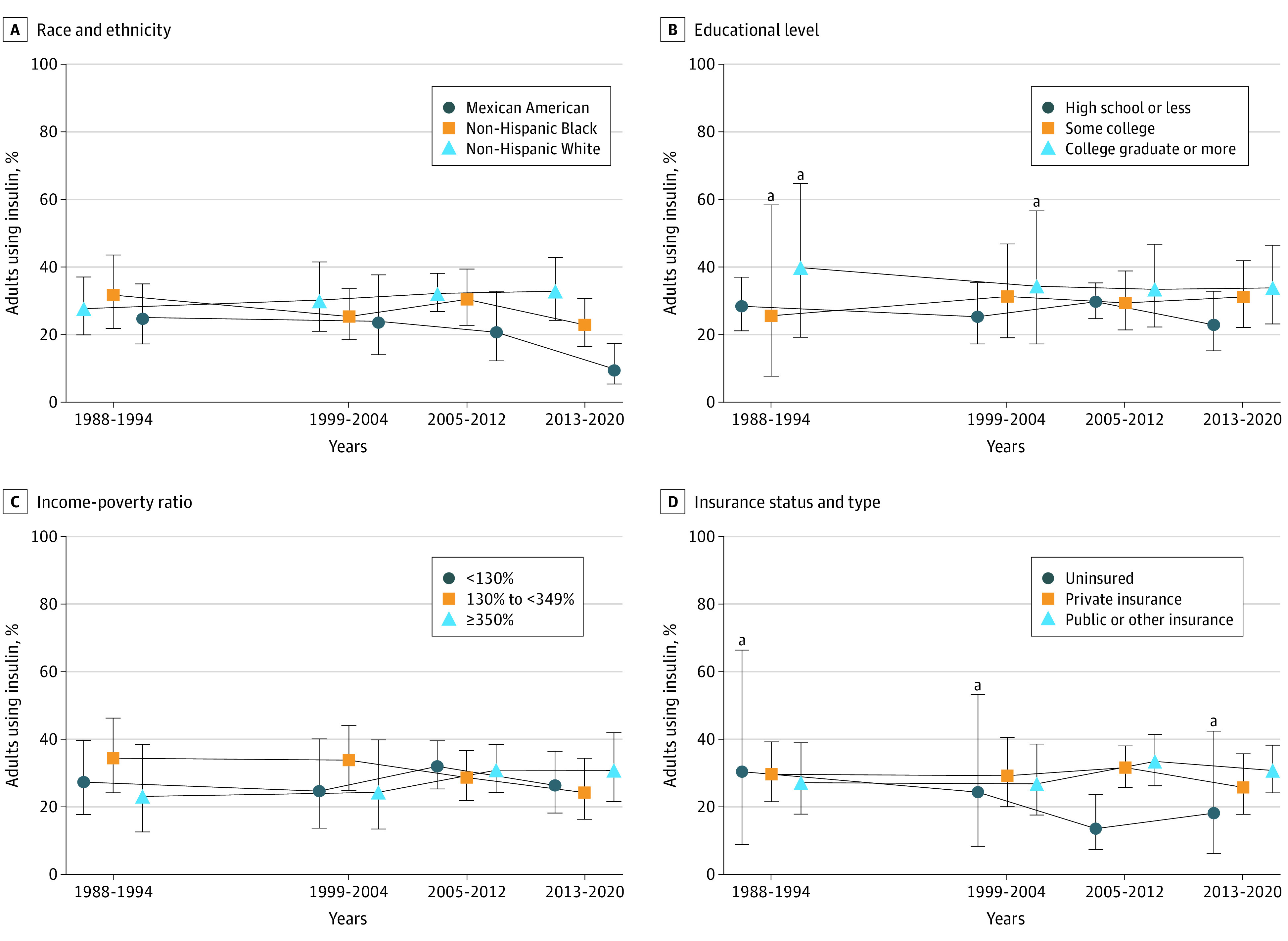
Trends in Glycemic Control Among US Adults With Diabetes Using Insulin From 1988 to 2020 Data are from the 1988 to 2020 National Health and Nutrition Examination Survey (N = 2482). Data were weighted to be nationally representative. Glycemic control was defined as a glycated hemoglobin (HbA_1c_) level less than 7% (to convert percentage of total hemoglobin to proportion of total hemoglobin, multiply by 0.01; to convert to millimoles per mole, multiply by 10.93 and subtract by 23.50). Adults who responded “other” for race and ethnicity were excluded in the race and ethnicity analysis but were included for all other subgroups. Error bars indicate 95% CIs. ^a^Estimate has a relative SE of 30% or greater and may be unreliable.

Trends in glycemic control were largely consistent across subgroups with the exception of race and ethnicity (Figure 2). Glycemic control decreased significantly for Mexican American adults using insulin (25.1% [95% CI, 17.2%-35.1%] in 1988-1994 to 9.9% [95% CI, 5.4%-17.4%] in 2013-2020; *P* = .004 for trend). In 2013-2020, non-Hispanic White individuals (32.9%; 95% CI, 24.3%-42.8%) and college-educated adults (33.9%; 95% CI, 23.3%-46.5%) had higher levels of glycemic control than their respective counterparts (eTable 2 in Supplement 1).

**Figure 3.**
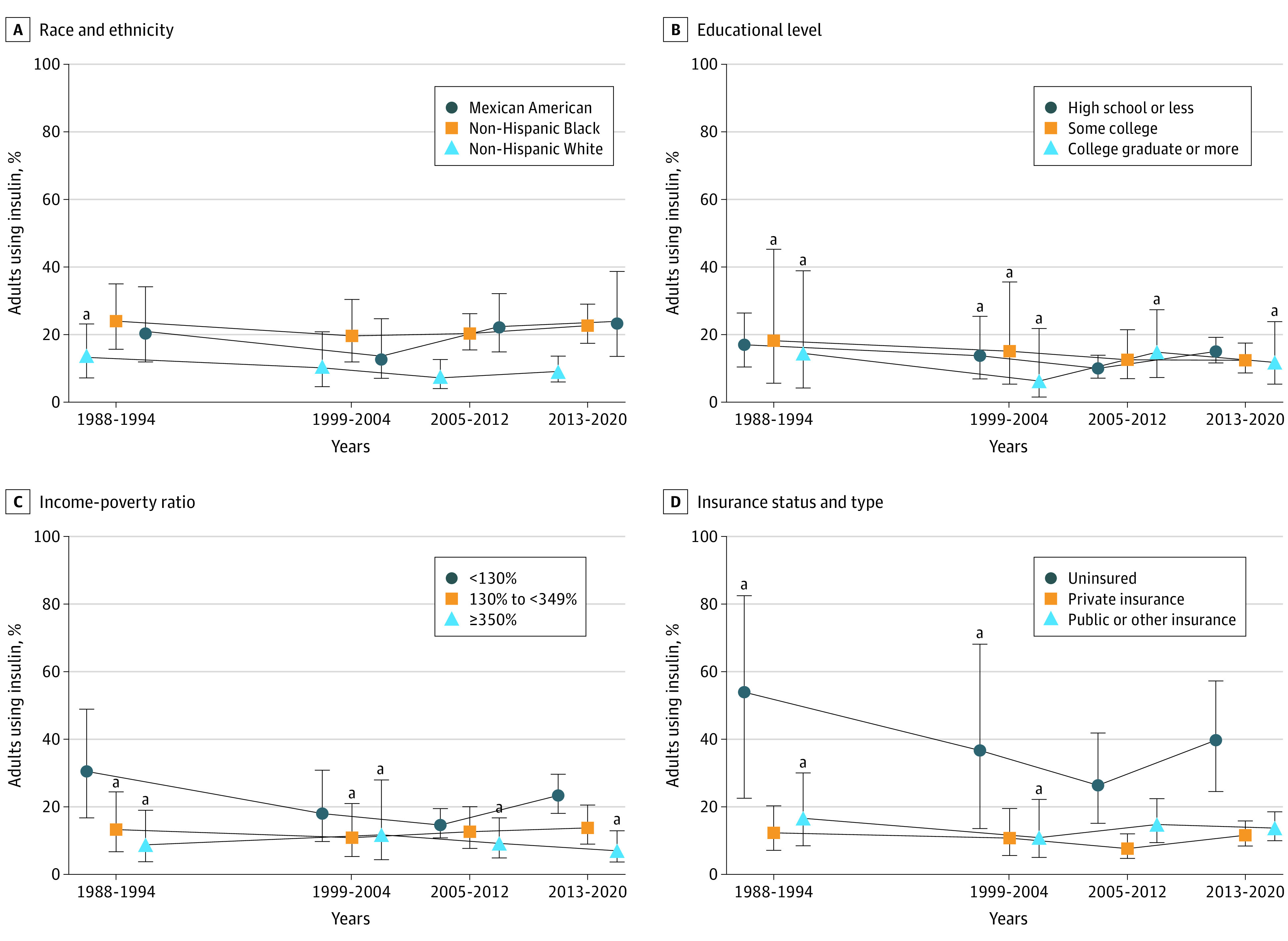
Trends in Severe Hyperglycemia Among US Adults With Diabetes Using Insulin From 1988 to 2020 Data are from the 1988 to 2020 National Health and Nutrition Examination Survey (N = 2482). Data were weighted to be nationally representative. Severe hyperglycemia was defined as a glycated hemoglobin level greater than 10% (to convert percentage of total hemoglobin to proportion of total hemoglobin, multiply by 0.01; to convert to millimoles per mole, multiply by 10.93 and subtract by 23.50). Adults who responded “other” for race and ethnicity were excluded in the race and ethnicity analysis but were included for all other subgroups. Error bars indicate 95% CIs. ^a^Estimate has a relative SE of 30% or greater and may be unreliable.

Severe hyperglycemia (HbA_1c_ level >10%) remained largely unchanged for all subgroups (Figure 3). In 2013-2020, the prevalence of severe hyperglycemia was roughly twice as high for Mexican Americans (23.9%; 95% CI, 13.6%-38.7%) and non-Hispanic Black adults (22.7%; 95% CI, 17.4%-29.0%) than it was for non-Hispanic White adults (9.1%; 95% CI, 6.0%-13.7%). Adults who had a income-poverty ratio less than 130% of the FPL (23.4%; 95% CI, 18.1%-29.7%) and were uninsured (39.7%; 95% CI, 24.5%-57.2%) also had a higher prevalence of severe hyperglycemia compared with their counterparts (eTable 2 in Supplement 1).

Trends were similar when using alternate uniform HbA_1c_ cutoffs (HbA_1c_ level <8% and HbA_1c_ level >9%), age-adjusted estimates, and age-specific cutoffs for glycemic control and severe hyperglycemia (eTable 3 and eFigures 1 and 2 in Supplement 1). Results were also similar after excluding participants who may have had type 1 diabetes (eTable 4 and eFigures 3 and 4 in Supplement 1).

### Adjusted Likelihood of Achieving Glycemic Control

After adjusting for age, gender, race and ethnicity, education, and income, Mexican American adults using insulin were significantly less likely (odds ratio [OR], 0.45; 95% CI, 0.30-0.68) to achieve glycemic control than were non-Hispanic White adults (Table 2). Non-Hispanic Black adults (OR, 2.48; 95% CI, 1.71-3.61) and Mexican American adults (OR, 2.29; 95% CI, 1.32-3.98) using insulin were more likely to have severe hyperglycemia compared with non-Hispanic White adults. Adults aged 65 years or older were more likely to achieve glycemic control (OR, 1.71; 95% CI, 1.09-2.67) and less likely to have severe hyperglycemia (OR, 0.20; 95% CI, 0.12-0.32) than were adults in younger age categories.

**Table 2.** Age-, Gender-, Race-, Education-, and Income-Adjusted Factors Associated With Glycemic Control and Severe Hyperglycemia in NHANES From 1988 to 2020^a^

Sociodemographic variable	Odds ratio (95% CI)	
	Glycemic control	Severe hyperglycemia
Age category, y		
20-49	1 [Reference]	1 [Reference]
50-64	1.07 (0.69-1.65)	0.41 (0.27-0.62)
≥65	1.71 (1.09-2.67)	0.20 (0.12-0.32)
Gender		
Men	1 [Reference]	1 [Reference]
Women	0.80 (0.62-1.02)	1.27 (0.88-1.85)
Race and ethnicity^b^		
Mexican American	0.45 (0.30-0.68)	2.29 (1.32-3.98)
Non-Hispanic Black	0.85 (0.61-1.19)	2.48 (1.71-3.61)
Non-Hispanic White	1 [Reference]	1 [Reference]
BMI		
<25	1 [Reference]	1 [Reference]
25 to <30	0.90 (0.56-1.46)	0.88 (0.49-1.57)^c^
≥30	0.92 (0.56-1.50)	0.75 (0.47-1.30)
Educational level		
High school or less	1 [Reference]	1 [Reference]
Some college	1.29 (0.90-1.85)	0.75 (0.48-1.19)
College graduate or above	1.43 (0.94-2.18)	0.87 (0.47-1.63)^c^
Insurance status		
Uninsured	1 [Reference]	1 [Reference]
Insured	1.38 (0.74-2.58)^c^	0.38 (0.24-0.62)
Private insurance	1.26 (0.66-2.41)^c^	0.35 (0.21-0.58)
Public or other insurance	1.60 (0.84-3.03)^c^	0.44 (0.26-0.74)
Hospital visits in 1 y, No.		
0	1 [Reference]	1 [Reference]
1	1.15 (0.39-3.42)^c^	2.14 (0.57-8.00)^c^
2-3	0.67 (0.29-1.53)^c^	1.26 (0.43-3.69)^c^
≥4	0.80 (0.36-1.76)^c^	0.93 (0.33-2.62)^c^
Immigration status		
US-born	1 [Reference]	1 [Reference]
Not US-born	1.49 (0.73-3.04)^c^	0.77 (0.48-1.24)
Income-poverty ratio		
<130% of FPL	1 [Reference]	1 [Reference]
130%-349% of FPL	0.83 (0.56-1.24)	0.68 (0.44-1.06)
≥350% of FPL	0.78 (0.53-1.13)	0.40 (0.23-2.29)
Duration of diabetes, y		
<5	1 [Reference]	1 [Reference]
5-15	0.70 (0.45-1.10)	0.87 (0.51-1.49)
>15	0.70 (0.47-1.03)	0.70 (0.39-1.25)

Abbreviations: BMI, body mass index (calculated as weight in kilograms divided by height in meters squared); FPL, federal poverty level; HbA_1c_, glycated hemoglobin; NHANES, National Health and Nutrition Examination Survey.

SI conversion factor: To convert percentage of total hemoglobin to proportion of total hemoglobin, multiply by 0.01; to convert to millimoles per mole, multiply by 10.93 and subtract by 23.50.

^a^ We excluded 351 respondents who were missing data on these variables. This population includes 2131 adults with diabetes who were using insulin. Glycemic control was defined as an HbA_1c_ level less than 7%, and severe hyperglycemia as an HbA_1c_ level greater than 10%.

^b^ Adults who responded “other” for race and ethnicity were excluded in the race and ethnicity analysis.

^c^ Estimate has a relative SE of 30% or greater and may be unreliable.

## Discussion

From 1988-1994 to 2013-2020, there was no significant change in the percentage of adults using insulin or the prevalence of glycemic control and severe hyperglycemia among US adults with diabetes using insulin. Overall, less than 30% of patients with diabetes using insulin had an HbA_1c_ level less than 7%, while approximately 15% had an HbA_1c_ level greater than 10%.

Few population-based studies have examined glycemic control among patients using insulin. Previous work showed that the prevalence of glycemic control did not change among US adults with diabetes using insulin from 1988 to 2012, averaging 33% throughout the study period.^8^ Our updated results, based on nationally representative data collected from 1988 to 2020, extend this work and show that glycemic control continued to stagnate among insulin users. Our results also establish that the prevalence of severe hyperglycemia did not decrease over time.^16^

Several factors may have contributed to the lack of improvement in glycemic control. First, the rising cost of insulin is likely leading to medication nonadherence.^17^ Approximately one-third of US adults using insulin report either rationing, dose skipping, or delaying prescription refills to save money.^18^ Second, only a small proportion of practitioners may be starting or intensifying insulin therapy in a timely manner.^19^ Third, acceptability of insulin remains low among patients, leading to reluctance to begin or continue using insulin therapy as recommended.^20^

Trends in glycemic control varied considerably across race and ethnicity. While glycemic control was stable for non-Hispanic White adults using insulin, we found that control declined significantly among Mexican American adults. These disparities may be driven in part by differences in socioeconomic resources, though differences persisted in analyses that adjusted for educational level. Other potential contributors may include unique cultural factors and health beliefs (eg, fear of needles), slower treatment intensification, differences in health care literacy, and discrimination.^21,22^ Improving diabetes care among Mexican American adults may require culturally tailored interventions.

Hyperglycemic emergencies have increased significantly since the mid-2000s and are especially common in patients with low income and racial and ethnic minority patients.^23,24^ Our results suggest that suboptimal use of insulin may partly drive these trends. Among adults using insulin, we found that racial and ethnic minority patients, uninsured patients, and those with low family income had the highest levels of severe hyperglycemia. These results suggest that addressing barriers to insulin therapy may be important for reducing hyperglycemic crises in high-risk populations.

This is one of the first nationally representative studies to characterize glycemic control and severe hyperglycemia among US adults with diabetes using insulin. We analyzed the most recent national data available in a large sample of adults with diagnosed diabetes. Nearly 3 decades of data were collected using rigorous and standardized protocols.

### Limitations

Certain limitations should be considered in the interpretation of our results. First, this analysis used cross-sectional data, and we could not determine the causes underlying the trends in glycemic control. Second, use of insulin was self-reported and did not include information on insulin type, dosage, or adherence. Third, to maximize sample size and precision, other concomitant therapies that may influence HbA_1c_ levels were not explored. Fourth, the NHANES only sampled noninstitutionalized adults, and therefore, certain segments of the population with diabetes are not represented in these estimates. Fifth, due to the relatively small sample size, the study power was insufficient to detect small to moderate changes in insulin use or glycemic control without pooling survey years.

## Conclusions

This serial cross-sectional study of NHANES data from 1988 to 2020 demonstrated that despite advancements in insulin formulations and diabetes management strategies, glycemic control and severe hyperglycemia among adults using insulin did not improve in the general US adult population with diabetes. Racial and ethnic disparities in glycemic control among adults with type 2 diabetes persisted and increased. Current rates of glycemic control in minority groups, especially Mexican American individuals, remain unacceptably low. Efforts to facilitate access to insulin will be critical to improve glycemic management. Addressing clinical inertia among practitioners and improving the care process may optimize glycemic control among patients using insulin.

## Notes

Supplement 1. **eTable 1.** Trends in Insulin Use Among US Adults With Diabetes, NHANES 1988-2020 **eTable 2.** Trends in Glycemic Control (HbA1c <7%) and Severe Hyperglycemia (HbA1c >10%) Among Adults With Diabetes Using Insulin, NHANES 1988-2020 **eTable 3.** Glycemic Control Among Adults With Diabetes Using Insulin, Unadjusted and Age-adjusted Based on Age Cutoff Recommendations **eFigure 1.** Trends in Glycemic Control (HbA_1c_<8%) Among Adults with Diabetes Using Insulin **eFigure 2.** Trends in Glycemic Control (HbA1c >9%) Among Adults With Diabetes Using Insulin **eTable 4.** Characteristics of Adults Diagnosed With Type 2 Diabetes Using Insulin, HNANES, 1988-2020 **eFigure 3.** Trends in Glycemic Control (HbA1c <7%) Among Adults With Type 2 Diabetes Using Insulin **eFigure 4.** Trends in Severe Hyperglycemia (HbA_1c_>10%) Among Adults With Type 2 Diabetes Using Insulin

Supplement 2.
Data Sharing Statement
