# Supplementary material for: Trends and Disparities in Glycemic Control and Severe Hyperglycemia Among US Adults With Diabetes Using Insulin, 1988-2020
Source: JAMA Netw Open. 2022 Dec 20;5(12):e2247656. doi: 10.1001/jamanetworkopen.2022.47656 (PMC9856837; doi:10.1001/jamanetworkopen.2022.47656)
Supplement: Supplement 1. — eTable 1. Trends in Insulin Use Among US Adults With Diabetes, NHANES 1988-2020 eTable 2. Trends in Glycemic Control (HbA1c <7%) and Severe Hyperglycemia (HbA1c >10%) Among Adults With Diabetes Using Insulin, NHANES 1988-2020 eTable 3. Glycemic Control Among Adults With Diabetes Using Insulin, Unadjusted and Age-adjusted Based on Age Cutoff Recommendations eFigure 1. Trends in Glycemic Control (HbA1c<8%) Among Adults with Diabetes Using Insulin eFigure 2. Trends in Glycemic Control (HbA1c >9%) Among Adults With Diabetes Using Insulin eTable 4. Characteristics of Adults Diagnosed With Type 2 Diabetes Using Insulin, HNANES, 1988-2020 eFigure 3. Trends in Glycemic Control (HbA1c <7%) Among Adults With Type 2 Diabetes Using Insulin eFigure 4. Trends in Severe Hyperglycemia (HbA1c>10%) Among Adults With Type 2 Diabetes Using Insulin [file jamanetwopen-e2247656-s001.pdf]

Supplementary Online Content

Venkatraman S, Echouffo-Tcheugui JB, Selvin E, Fang M. Trends and disparities in glycemic control and severe hyperglycemia among US adults with diabetes using insulin, 1988-2020. *JAMA Netw Open*. 2022;5(12):e2247656. doi:10.1001/jamanetworkopen.2022.47656

eTable 1. Trends in Insulin Use Among US Adults With Diabetes, NHANES 1988-2020
eTable 2. Trends in Glycemic Control (HbA1c <7%) and Severe Hyperglycemia (HbA1c >10%) Among Adults With Diabetes Using Insulin, NHANES 1988-2020
eTable 3. Glycemic Control Among Adults With Diabetes Using Insulin, Unadjusted and Age-adjusted Based on Age Cutoff Recommendations
eFigure 1. Trends in Glycemic Control (HbA_{1c} <8%) Among Adults with Diabetes Using Insulin
eFigure 2. Trends in Glycemic Control (HbA1c >9%) Among Adults With Diabetes Using Insulin
eTable 4. Characteristics of Adults Diagnosed With Type 2 Diabetes Using Insulin, NHANES 1988-2020
eFigure 3. Trends in Glycemic Control (HbA1c <7%) Among Adults With Type 2 Diabetes Using Insulin
eFigure 4. Trends in Severe Hyperglycemia (HbA_{1c} >10%) Among Adults With Type 2 Diabetes Using Insulin

This supplementary material has been provided by the authors to give readers additional information about their work.

© 2022 Venkatraman S et al. *JAMA Network Open*.

eTable 1: Trends in Insulin Use Among US Adults With Diabetes, NHANES 1988-2020

**Percentage**	**1988-1994**	**1999-2004**	**2005-2012**	**2013-2020**	**P-Trend**
Overall % (Weighted)	30.5%	25.7%	29.2%	28.2%	0.81
Unweighted Number	475	388	755	864	

© 2022 Venkatraman S et al. *JAMA Network Open*.

eTable 2: Trends in Glycemic Control (HbA_{1c} <7%) and Severe Hyperglycemia (HbA_{1c} >10%) Among Adults With Diabetes Using Insulin, NHANES 1988-2020

**Glycemic Control (A1c<7%)**				
**Characteristics**	**1988-1994 % (95% CI)**	**1999-2004 % (95% CI)**	**2005-2012 % (95% CI)**	**2013-2020 % (95% CI)**
**Overall**	29.2 (22.6–36.8)	28.0 (21.5–35.7)	30.4 (26.1–35.1)	27.5 (21.7–34.2)
**Race/Ethnicity**				
Mexican American	25.1 (17.2–35.1)	23.9 (14.0–37.8)	20.7 (12.2–32.9)	9.9 (5.4–17.4)
Non-Hispanic Black	31.8 (21.9–43.6)	25.4 (18.6–33.6)	30.4 (22.7–39.4)	22.9 (16.6–30.7)
Non-Hispanic White	27.7 (19.9–37.1)	30.3 (21.0–41.5)	32.3 (26.9–38.1)	32.9 (24.3–42.8)
**Education**				
High School or less	28.5 (21.2–37.1)	25.3(17.3–35.5)	29.8 (24.8–35.4)	22.9 (15.3–32.9)
Some College	25.6 (7.8–58.5)	31.4 (19.1–46.9)	29.5 (21.5–38.9)	31.2 (22.2–41.9)
College Graduate or Above	39.9 (19.3–64.8)	34.4 (17.3–56.7)	33.5 (22.4–46.8)	33.9 (23.2–46.5)
**Income-Poverty Ratio**				
<130%	27.4 (17.8–39.7)	24.7 (13.8–40.2)	32.1 (25.3–39.6)	26.4 (18.3–36.5)
130-349%	34.5 (24.3–46.4)	33.9 (24.9–44.2)	28.7 (21.9–36.7)	24.3 (16.4–34.5)
≥350%	23.2 (12.7–38.6)	24.4 (13.5–39.9)	30.9 (24.3–38.5)	30.9 (21.6–42.0)
**Insurance Status**				
Uninsured	30.5 (8.8–66.4)	24.4 (8.4–53.3)	13.6 (7.4–23.7)	18.1 (6.2–42.4)
Private Insurance	29.7 (21.6–39.3)	29.3 (20.1–40.6)	31.6 (25.8–38.1)	25.8 (17.8–35.8)
Public/Other Insurance	27.2 (17.9–39.0)	26.8 (17.6–38.7)	33.5 (26.3–41.5)	30.8 (24.4–38.3)
**Severe Hyperglycemia (A1c>10%)**				
**Characteristics**	**1988-1994 % (95% CI)**	**1999-2004 % (95% CI)**	**2005-2012 % (95% CI)**	**2013-2020 % (95% CI)**
**Overall**	18.2 (13.0–24.8)	14.0 (8.9–21.4)	14.6 (10.8–19.6)	14.6 (12.0–17.5)
**Race/Ethnicity**				
Mexican American	21.0 (12.0–34.2)	13.6 (7.1–24.7)	22.4 (15.0–32.1)	23.9 (13.6–38.7)
Non-Hispanic Black	24.1 (15.7–35.0)	19.6 (12.0–30.4)	20.3 (15.5–26.2)	22.7 (17.4–29.0)
Non-Hispanic White	13.3 (7.2–23.2)	10.2 (4.6–20.9)	7.2 (4.0–12.7)	9.1 (6.0–13.7)
**Education**				
High School or less	17.1 (10.6–26.5)	13.9 (7.0–25.5)	10.2 (7.3–14.0)	15.1 (11.8–19.3)
Some College	18.3 (5.7–45.3)	15.2 (5.5–35.7)	12.7 (7.1–21.6)	21.5 (8.8–17.6)
College Graduate or Above	14.6 (4.4–39.0)	6.4 (1.6–21.9)	14.9 (7.5–27.5)	11.9 (5.5–23.9)
**Income-Poverty Ratio**				
<130%	30.5 (16.8–48.9)	18.0 (9.8–30.9)	14.7 (10.9–19.5)	23.4 (18.1–29.7)
130-349%	13.3 (6.8–24.4)	10.9 (5.3–21.0)	12.7 (7.8–20.0)	13.8 (9.0–20.6)
≥350%	8.8 (3.8–19.0)	11.8 (4.4–28.0)	9.2 (4.9–16.8)	7.0 (3.7–13.0)
**Insurance Status**				
Uninsured	53.9 (22.6–82.4)	36.7 (13.6–68.1)	26.4 (15.1–41.8)	39.7 (24.5–57.2)
Private Insurance	12.3 (7.2–20.3)	10.7 (5.6–19.5)	7.7 (4.8–12.1)	11.6 (8.4–15.8)
Public/Other Insurance	16.6 (8.5–30.0)	10.9 (5.0–22.2)	14.8 (9.4–22.4)	13.7 (10.0–18.5)

© 2022 Venkatraman S et al. *JAMA Network Open*.

eTable 3: Glycemic Control Among Adults With Diabetes Using Insulin, Unadjusted and Age-adjusted Based on Age Cutoff Recommendations.

**Prevalence**	**1988-1994**	**1999-2004**	**2005-2012**	**2013-2020**	**P-Trend**
Unadjusted	29.2 (22.6–36.8)	28.0 (21.5–35.7)	30.4 (26.1–35.1)	27.5 (21.7–34.2)	0.87
Age-adjusted	29.2 (22.7–36.7)	28.3 (21.9–35.6)	30.8 (26.4–35.4)	27.4 (21.5–34.2)	0.92
Age-specific cutoff at 75 years[a]	30.8 (24.0–38.6)	28.7 (22.4–36.0)	34.6 (30.2–39.2)	31.7 (25.9–38.2)	0.39
Age-specific cutoff at 65 and 75 years[b]	35.3 (27.7–43.8)	30.7 (24.5–37.7)	38.4 (33.7–43.2)	36.4 (30.1–43.3)	0.35

[a] Age-specific A1c goals specified by the ADA 2022 Standard of Care of <7% (53 mmol/mol) for nonpregnant adults and <8% (64 mmol/mol) for patients with “limited life expectancy”. This was chosen to be an age of 75 years in other literature and for our study.

[b] Age-specific A1c goal of <7% (53 mmol/mol) for nonpregnant adults younger than 65 years, a goal of <7.5% (58 mmol/mol) for adults aged 65-74 years, and a goal of <8% (64 mmol/mol) for adults aged 75 years and older.

© 2022 Venkatraman S et al. *JAMA Network Open*.

eFigure 1: Trends in Glycemic Control (HbA$_{1c}$ <8%) Among Adults With Diabetes Using Insulin, (A) Overall, (B) Race/Ethnicity, (C) Education, (D) Poverty-Income Ratio, (E) Insurance status, the National Health and Nutrition Examination Survey, 1988 to 2020, N = 2,482[a]

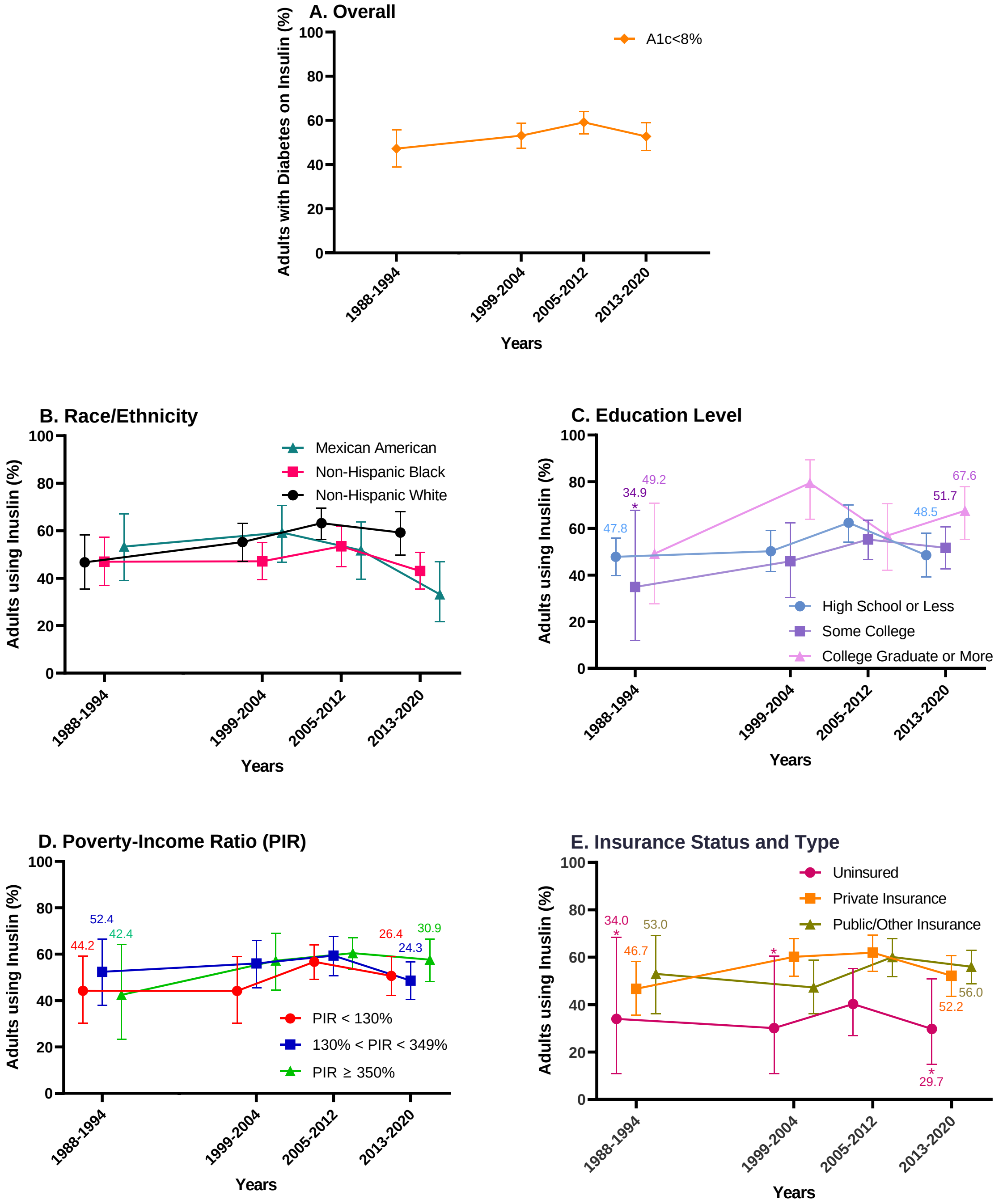

[a] Adults who responded "Other" were excluded in the race/ethnicity analysis but were included for all other subgroups.

© 2022 Venkatraman S et al. *JAMA Network Open*.

eFigure 2: Trends in Glycemic Control (HbA_{1c} >9%) Among Adults With Diabetes Using Insulin, (A) Overall, (B) Race/Ethnicity, (C) Education, (D) Poverty-Income Ratio, (E) Insurance status, the National Health and Nutrition Examination Survey, 1988 to 2020, N = 2,482[a]

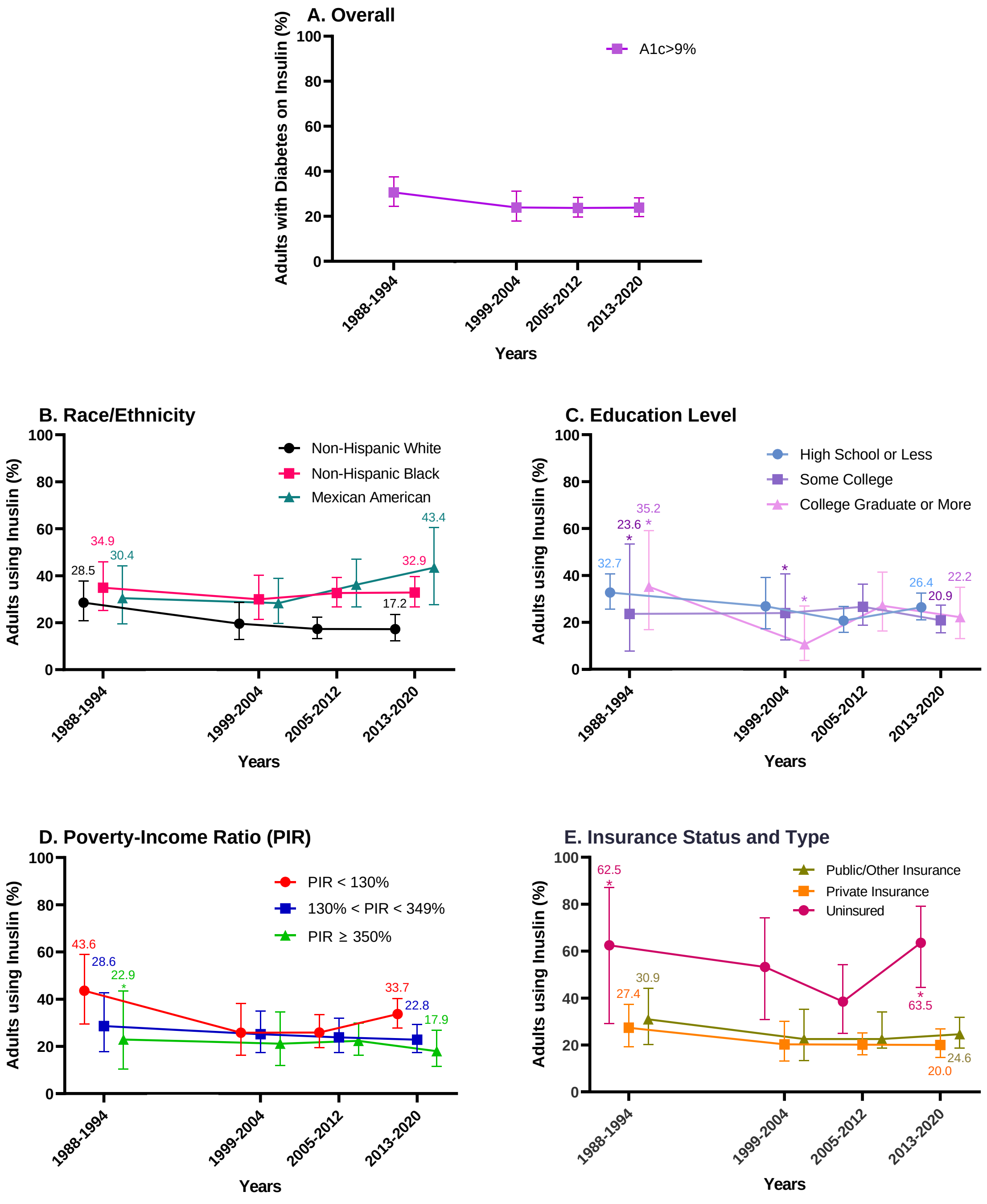

[a] Adults who responded "Other" were excluded in the race/ethnicity analysis but were included for all other subgroups.

© 2022 Venkatraman S et al. *JAMA Network Open*.

eTable 4: Characteristics of Adults Diagnosed With Type 2 Diabetes Using Insulin, National Health and Nutrition Examination Survey, 1988-2020, N = 2,386[a]

Characteristics	1988-1994 (n= 458)	1999-2004 (n= 374)	2005-2012 (n= 716)	2013-2020 (n= 838)
**Mean Age**	63.1 (60.8–65.5)	60.5 (58.3–62.7)	60.8 (59.4–62.2)	61.7 (60.2–63.1)
**Age Categories**				
20 to 49	15.8 (9.1–25.9)	19.7 (14.2–26.5)	19.2 (15.3–23.8)	18.5 (15.1–22.3)
50 to 64	32.7 (26.9–39.1)	34.9 (28.5–42.0)	39.6 (35.2–44.1)	36.3 (31.9–41.0)
≥65	51.5 (44.2–58.8)	45.3 (38.4–52.4)	41.2 (36.8–45.8)	45.2 (40.0–50.6)
**Sex**				
Male	36.2 (29.0–44.0)	45.3 (38.3–52.4)	51.2 (46.8–55.6)	53.9 (48.2–59.4)
Female	63.8 (56.0–71.0)	54.7 (47.6–61.7)	48.8 (44.4–53.2)	46.1 (40.6–51.8)
**Race/Ethnicity**				
Mexican American	4.7 (3.6–6.3)	5.3 (3.3–8.4)	7.0 (4.4–10.8)	9.2 (6.8–12.3)
Non-Hispanic Black	23.1 (18.3–28.8)	20.2 (13.7–28.8)	20.4 (16.4–25.1)	15.3 (12.1–19.1)
Non-Hispanic White	68.8 (62.3–74.6)	64.2 (55.8–71.9)	63.3 (57.0–69.3)	62.3 (56.7–67.6)
**Body Mass Index (BMI)**				
Mean BMI (kg/m^2)	30.7 (29.6–31.8)	32.7 (31.5–34.0)	34.5 (33.7–35.3)	33.7 (32.9–34.4)
<25	20.6 (14.2–29.0)	15.1 (10.0–22.2)	8.5 (6.4–11.1)	8.1 (6.0–10.7)
25-29	36.6 (28.7–45.2)	20.9 (17.0–25.5)	19.6 (15.8–24.0)	23.5 (18.9–28.9)
>30	42.8 (35.5–50.5)	64.0 (57.3–70.1)	71.9 (67.3–76.1)	68.4 (62.8–73.5)
**Education**				
College or Above	81.3 (73.0–87.5)	65.2 (58.8–71.2)	52.3 (46.4–58.1)	52.4 (47.6–57.2)
Some College	10.3 (5.9–17.1)	22.5 (16.6–29.6)	31.1 (26.4–36.2)	31.4 (27.3–35.8)
High School or less	8.4 (5.3–13.1)	12.3 (8.5–17.5)	16.6 (12.3–22.0)	16.2 (12.9–20.1)
**Insurance Status**				
Insured	93.1 (86.8–96.6)	91.3 (85.4–94.9)	91.5 (88.2–94.0)	95.7 (93.9–97.0)
Private Insurance	68.1 (58.3–76.6)	54.8 (47.3–62.0)	54.5 (49.1–59.7)	52.1 (46.9–57.3)
Public/Other Insurance	25.0 (18.9–32.3)	36.5 (31.1–42.2)	37.1 (32.1–42.3)	43.6 (38.5–48.8)
Uninsured	6.9 (3.4–13.2)	8.7 (5.1–14.6)	8.5 (6.0–11.8)	4.3 (3.0–6.1)
**Number of Hospital Visits**				
None	2.6 (1.2–5.6)	No Observations	1.8 (0.7–4.2)	0.9 (0.6–1.5)
1	5.8 (3.3–10.1)	3.6 (1.7–7.6)	2.9 (1.5–5.3)	3.1 (1.8–5.4)
2-3	16.8 (12.5–22.1)	17.1 (12.2–23.5)	13.8 (10.8–17.4)	19.9 (15.8–24.8)
4+	74.8 (69.5–79.5)	79.3 (72.6–4.6)	81.6 (77.6–85.0)	76.0 (70.8–80.5)
**Immigration Status**				
US-Born	94.1 (90.2–96.4)	89.3 (84.8–92.7)	90.5 (87.9–92.5)	84.6 (81.1–87.6)
Not US-Born	5.9 (3.6–9.8)	10.7 (7.3–15.2)	9.5 (7.5–12.1)	15.4 (12.4–18.9)
**Income-Poverty Ratio**				
Mean PIR	2.4 (2.1–2.8)	2.4 (2.2–2.6)	2.6 (2.4–2.7)	2.6 (2.4–2.9)
<130% of the FPL	26.3 (19.6–34.3)	27.7 (22.3–33.9)	24.9 (20.9–29.4)	28.2 (23.5–33.4)
130%–349% of the FPL	46.4 (39.2–53.9)	37.7 (32.0–43.9)	38.3 (33.4–43.4)	32.9 (27.2–39.1)
≥350% of the FPL	27.3 (21.2–34.3)	34.5 (28.4–41.2)	36.8 (32.3–41.6)	38.9 (31.5–46.8)
**Calibrated A1c (%)**	8.1 (7.9–8.4)	8.2 (7.9–8.5)	8.0 (7.9–8.2)	8.1 (7.9–8.3)
**Duration of Diabetes (Yr.)**	12.8 (11.2–14.4)	16.7 (14.9–18.6)	16.3 (15.2–17.4)	17.1 (16.2–18.1)

[a] We excluded 96 participants who may have type 1 diabetes, defined as diagnosed with diabetes at age 30 or under, currently taking only insulin medication, and initiating insulin therapy within 1 year of their diabetes diagnosis.

© 2022 Venkatraman S et al. *JAMA Network Open*.

eFigure 3: Trends in Glycemic Control (HbA_{1c} <7%) Among Adults With Type 2 Diabetes Using Insulin, by (A) Race/Ethnicity, (B) Education, (C) Poverty-Income Ratio, (D) Insurance status, the National Health and Nutrition Examination Survey, 1988 to 2020, N = 2,458[a]

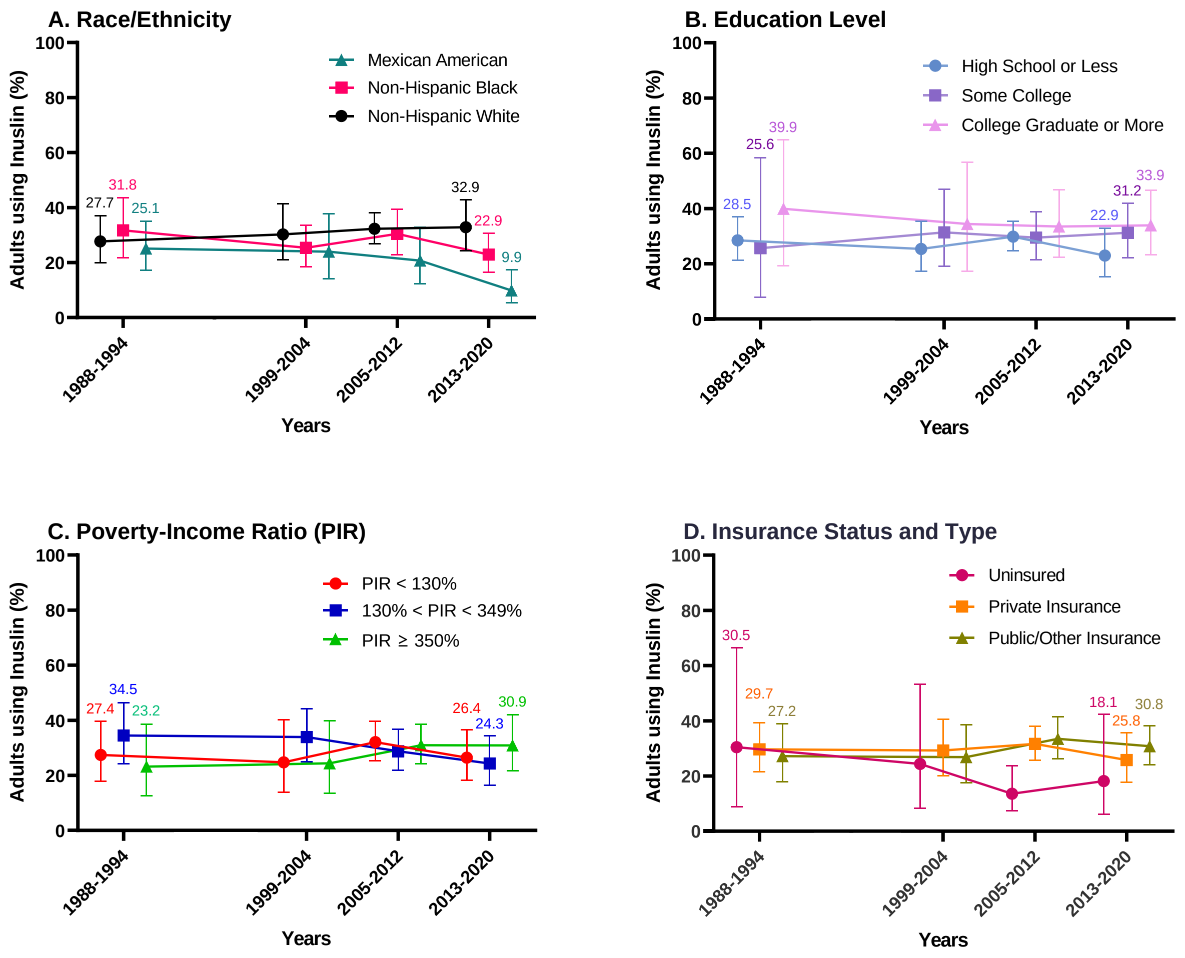

[a] We excluded 96 participants who may have type 1 diabetes, defined as diagnosed with diabetes at age 30 or under, currently taking only insulin medication, and initiating insulin therapy within 1 year of their diabetes diagnosis. Adults who responded "Other" were excluded in the race/ethnicity analysis but were included for all other subgroups.

© 2022 Venkatraman S et al. *JAMA Network Open*.

eFigure 4: Trends in Severe Hyperglycemia (HbA_{1c} >10%) Among Adults With Type 2 Diabetes Using Insulin, by (A) Race/Ethnicity, (B) Education, (C) Poverty-Income Ratio, (D) Insurance status, the National Health and Nutrition Examination Survey, 1988 to 2020, N = 2,458[a]

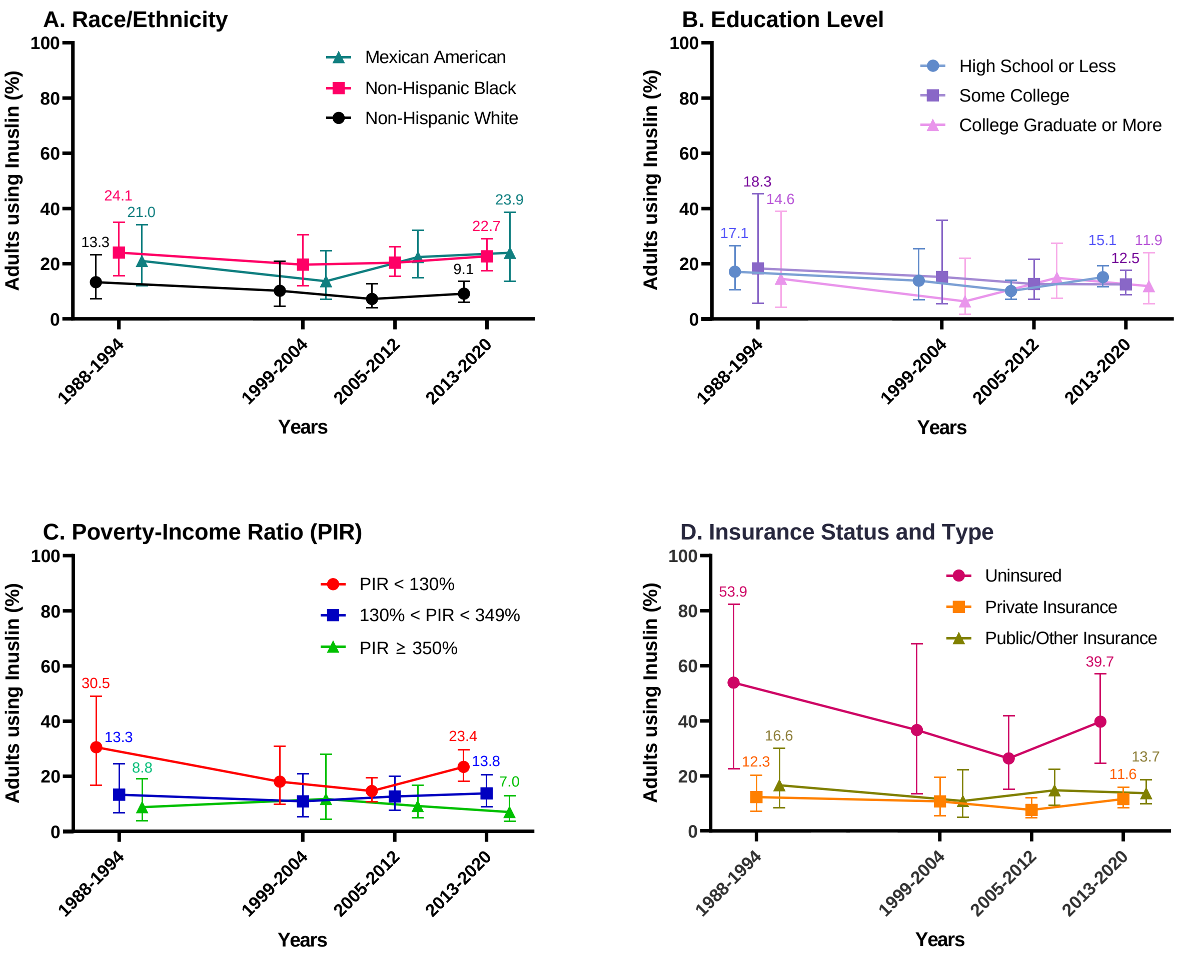

[a] We excluded 96 participants who may have type 1 diabetes, defined as diagnosed with diabetes at age 30 or under, currently taking only insulin medication, and initiating insulin therapy within 1 year of their diabetes diagnosis. Adults who responded "Other" were excluded in the race/ethnicity analysis but were included for all other subgroups.

© 2022 Venkatraman S et al. *JAMA Network Open*.
