# Supplementary material for: Trends and Disparities in Glycemic Control and Severe Hyperglycemia Among US Adults With Diabetes Using Insulin, 1988-2020
Source: JAMA Netw Open. 2022 Dec 20;5(12):e2247656. doi: 10.1001/jamanetworkopen.2022.47656 (PMC9856837; doi:10.1001/jamanetworkopen.2022.47656)
Supplement: Supplement 2. — Data Sharing Statement [file jamanetwopen-e2247656-s002.pdf]

Data Sharing Statement

Venkatraman. Trends and Disparities in Glycemic Control and Severe Hyperglycemia Among US Adults With Diabetes Using Insulin, 1988-2020. *JAMA Netw Open*. Published December 20, 2022. doi:10.1001/jamanetworkopen.2022.47656

Data
Data available: Yes
Data types: Deidentified participant data
How to access data: The NHANES database is publicly accessible and contains deidentified data for all participants utilized in this study. This dataset can be accessed, queried, and downloaded using this link: https://wwwn.cdc.gov/nchs/nhanes/Default.aspx
When available: With publication

Supporting Documents
Document types: None

Additional Information
Who can access the data: Since NHANES is public, the data is always available.
Types of analyses: Since NHANES is public, the data is always available for any project that requires it.
Mechanisms of data availability: The data is always publicly accessible and can be accessed at any time.
Any additional restrictions: None.
